# Unraveling yeast diversity in food fermentation using ITS1-2 amplicon-based metabarcoding

**DOI:** 10.1128/spectrum.00060-26

**Published:** 2026-07-06

**Authors:** Ines Pradal, Thomas Gettemans, Stefan Weckx

**Affiliations:** 1 Research Group of Industrial Microbiology and Food Biotechnology (IMDO), Faculty of Sciences and Bioengineering Sciences, Vrije Universiteit Brussel (VUB) 70493, Brussels, Belgium; University of Turin, Gruglisco, Turin, Italy

**Keywords:** metagenetics, yeasts, food fermentation, PacBio, ITS

## Abstract

A detailed characterization of the microbial ecosystem involved in the production processes of fermented foods is essential. Although fermented foods are an important part of the human diet and have seen an increasing interest nowadays, some challenges still need to be solved. Specifically, yeast identification through culture-independent methodologies is still limited to the genus level. Unlike bacterial species identifications, long-read sequencing technologies have barely been used for yeast species identification, and, to the best of the authors’ knowledge, it has not been validated with mock communities reflecting food fermentation processes yet. Therefore, in the current study, we present an amplicon-based metabarcoding approach targeting the full-length internal transcribed spacer (ITS) region comprising ITS1, the 5.8S rRNA gene, and ITS2, using the PacBio HiFi sequencing platform. This method was validated using DNA-based mock communities composed of yeast species involved in sourdough, lambic beer, and cocoa fermentation processes. Accurate species-level identification was achieved for most of the species. However, special attention should be given to *Saccharomyces*-rich niches, as accurate species-level identification for this genus is still challenging. Furthermore, underestimation of the relative abundance of species with short ITS regions, such as *Pichia* and *Brettanomyces*, occurred. In addition, the method was successfully applied to describe the yeast diversity present in two sourdough and two lambic beer samples. Overall, the current method provides an unprecedented way of determining the species-level yeast composition of complex ecosystems present in fermented food products.

**IMPORTANCE:**

To date, species-level identification of common yeasts present in food fermentation ecosystems has been difficult, if not impossible, when using short-read sequencing methods. However, species-level identification is essential when evaluating and describing the characteristics of fermented food microbiomes. The current study reports on the development and validation of an amplicon-based metabarcoding approach combined with long-read PacBio HiFi sequencing targeting the full internal transcribed spacer (ITS) region, comprising the ITS1 and ITS2 regions, as well as the 5.8S rRNA gene. The described methodology enables species-level identification of the most common yeasts present in food fermentation ecosystems. This new methodology provides an important tool not only for the investigation of fermented foods but also for other fields engaged in complex microbial community analysis.

## INTRODUCTION

Fermented foods and beverages have been an important part of the human diet for centuries and have seen an increasing interest nowadays (1). As a result of the great importance of fermented foods and beverages, studying the microbial ecosystem composition involved in their production processes in detail is essential. Fermented foods are produced through the metabolic activity of yeasts, lactic acid bacteria (LAB), coagulase-negative cocci, acetic acid bacteria, and/or filamentous fungi (1–3). Among the microorganisms involved, yeasts are key microorganisms during the production of sourdough, beer, and wine, among others, as well as during cocoa fermentation (2, 4).

Yeast diversity in food fermentation processes has traditionally been studied by culture-dependent approaches in the same way as is done for bacterial diversity (5). However, besides the benefit of being able to construct a collection of microbial strains for further investigation, those approaches are time-consuming and are biased by the selective growth media and conditions used (5, 6). The advances in high-throughput sequencing technologies allow the study of microbial communities using a culture-independent approach (5, 7). Specifically, amplicon-based metabarcoding, also known as metagenetics, has been the gold standard in this field, as it has a high throughput with relatively low costs (7–11). This technique consists of amplifying a specific genomic region expected to be present in all genomes of the microorganisms present in the ecosystem, followed by sequencing the amplicons obtained (7). Therefore, selecting an appropriate genomic region that enables a good taxonomic resolution is of crucial importance. Furthermore, selecting a proper sequencing technology is also of importance, as the technology chosen can also impose certain constraints. For example, Illumina MiSeq and Illumina NovaSeq are the most commonly used technologies, but they only allow for sequencing of short reads, despite their high accuracy. This short length results in a taxonomical resolution that seldom reaches the species level (10, 12). Long-read sequencing technologies, such as Pacific Biosciences’ (PacBio) HiFi long-read sequencing or Oxford Nanopore Technologies’ nanopore sequencing, are available on the market. The former one has emerged as a suitable technology for amplicon-based metabarcoding as it combines long reads with high accuracy (10, 13).

In the case of bacterial communities, the 16S rRNA gene is commonly considered to be the most suitable gene for identification (14, 15). Due to the length restriction imposed for Illumina sequencing, specific variable regions or combinations thereof, such as V1-V3, V4, and V3-V5, have been targeted (15–17). However, this only allowed for genus-level resolution (17, 18). Recently, PacBio’s HiFi sequencing allows the analysis of the full-length 16S rRNA gene (10, 15, 19, 20) and, therefore, a species-level resolution for most of the LAB commonly found during food fermentation. Specifically, it has been used to reveal the bacterial diversity of beer (21, 22), cheese (23, 24), and sourdough (25).

In the case of yeasts, there is no such gold standard. Variable regions of the 18S rRNA gene, the internal transcribed spacer 1 (ITS1) region, and the ITS2 region are some examples of the genomic regions targeted (9, 26–28). In all cases, the short length imposed by Illumina sequencing also leads to less accurate genus-level identification compared to long-read amplicon sequencing (29). In addition, comparisons of taxonomic assignment using different regions are available for specific samples, such as beer (27), wine (27), soil (9), human saliva (9), grape must (9), and bioaerosols (30). However, different microbial compositions are retrieved for different targeted regions, and the preferred region is environment-dependent. No consensus has been reached regarding which genomic region to target yet. Nevertheless, all studies pointed out the ITS regions as the primary fungal barcode because of a higher number of sequence variations (12, 31). Recently, PacBio HiFi sequencing of the genomic region comprising the ITS1, 5.8S rRNA gene, and ITS2 has been performed for metabarcoding of eukaryote-containing samples. Specifically, it has been used to describe the fungal diversity of coffee plant samples (32), wheat, maize, barley, and cover crop (clover, hairy vetch, oilseed radish, and fallow) samples (33), seawater samples (34), and soil samples (31, 35), with most studies focusing on filamentous fungi rather than yeasts. To the best of the authors’ knowledge, this method has barely been applied to the analysis of fermented food samples (21), nor has it been validated using mock communities reflecting food fermentation processes yet.

Hence, this study aimed to assess whether amplicon-based metabarcoding targeting the full ITS region, comprising the ITS1 and ITS2 regions as well as the 5.8S rRNA gene, enables species-level identification of the most common yeasts present in food fermentation ecosystems. After determining the appropriate primer sets and amplification conditions, the technique was tested using DNA-based mock communities (DbMC) representing the yeast composition of different fermentation stages of relevant fermented foods and beverages, namely sourdough, lambic beer, and cocoa. Finally, the fungal diversity of two sourdough samples and two lambic beer samples was assessed using this technique for validation.

## RESULTS

### Optimization of the PCR conditions: BITS-ITS4 vs ITS1-ITS4

PCR amplification using a general DbMC as DNA template and the BITS-ITS4 primer set (the BITS primer was extended with barcodes 04, 05, and 06; the ITS4 primer was extended with barcodes 19 and 20) resulted in no visible fragments on gel electrophoresis (data not shown). Hence, the primer combination BITS-ITS4 was deemed unsuccessful in amplifying the targeted microorganisms. On the contrary, the use of the ITS1-ITS4 primer set (the ITS1 primer was extended with barcodes 07, 08, and 09; the ITS4 primer was extended with barcodes 19 and 20) and the same DbMC as DNA template resulted in visually successful amplification. Specifically, the clearest bands were obtained when an annealing temperature of 52°C was used. The purification of the amplicons resulted in DNA concentrations between 4.9 and 49.0 ng/µL, with no relationship observed between the concentrations and primer-barcode combination or sample types. However, PCR amplifications involving the ITS1 primer extended with barcode 05 resulted in no amplification, most likely due to secondary structures formed between the primer and barcode sequences. Indeed, when checking the extended primer in OligoEvaluator, four possible moderate secondary structures were present involving the 3′ end of the primer, possibly preventing primer annealing.

### Sequencing data

An average of 118,998 reads per sample was obtained with a minimum of 9,718 reads and a maximum of 348,099 reads (Table S3). Those reads had an average length of 763 nucleotides, with 90% of the reads being between 600 and 900 nucleotides, while 10% of the reads were shorter than 600 nucleotides. Primer removal, quality filtering, and denoising resulted in the removal of 11% of the reads, yielding an average of 105,470 reads per sample, with a minimum of 8,007 reads (Lambic beer A) and a maximum of 310,563 reads (DbMC lambic beer equal 1). Most of the removed reads (8% of the total reads) were eliminated during the quality-filtering step. After data processing, the average length of the reads was 722 nucleotides, with a minimum length of 405 and a maximum length of 842 nucleotides. Only 15% of the reads were shorter than 600 bp.

Rarefaction analysis showed that all DbMCs and fermented food samples were sequenced with a sufficient depth to reliably assess the full fungal diversity (Fig. S1). The two DbMCs (cocoa equal 1 and cocoa late fermentation stages 3) with the highest number of species retrieved (16 species) reached a plateau (within 85,121 and 42,981 reads, respectively; Fig. S1A). In the case of the Lambic beer A and B samples, the plateau was reached with 4,741 and 5,301 reads, respectively, representing a maximum of 17 and 6 species, respectively (Fig. S1B). In the case of the Sourdough A and B samples, the plateau was reached with 43,521 and 8,041 reads, respectively, representing a maximum of 30 and 33 species, respectively.

### Taxonomic classification

#### Unidentifiable fungal genus reads

Taxonomical assignment at the species or genus level could not be achieved for all amplicon sequence variants (ASVs) obtained. The proportion of unidentifiable fungal genus reads (UFGRs) was small for the different DbMCs, with a maximal percentage of 0.2% of the total reads in the DbMC cocoa equal 3. The Lambic beer A and B samples displayed similarly low percentages of UFGR. On the contrary, the sourdough samples had large amounts of UFGR, with 16.7% and 60.8% in Sourdough A and B, respectively. Blastn searches of these ASVs revealed that 99.4% and 99.8% of the UFGR, respectively, aligned to the ITS region of *Simplicia felix* (87%–91% sequence identity and 85.6%–88.3% coverage). Furthermore, blastn searches of these ASVs against the genomes of the type material of *Triticum aestivum* (soft wheat) and *Secale cereale* (rye) resulted in alignments of the ASVs with the ITS regions of these two plant species, with 100% sequence identity and coverage. Therefore, the UFGRs were removed before calculating the relative abundances of fungal species in each sample.

#### DNA-based mock communities

**Fig 1:**
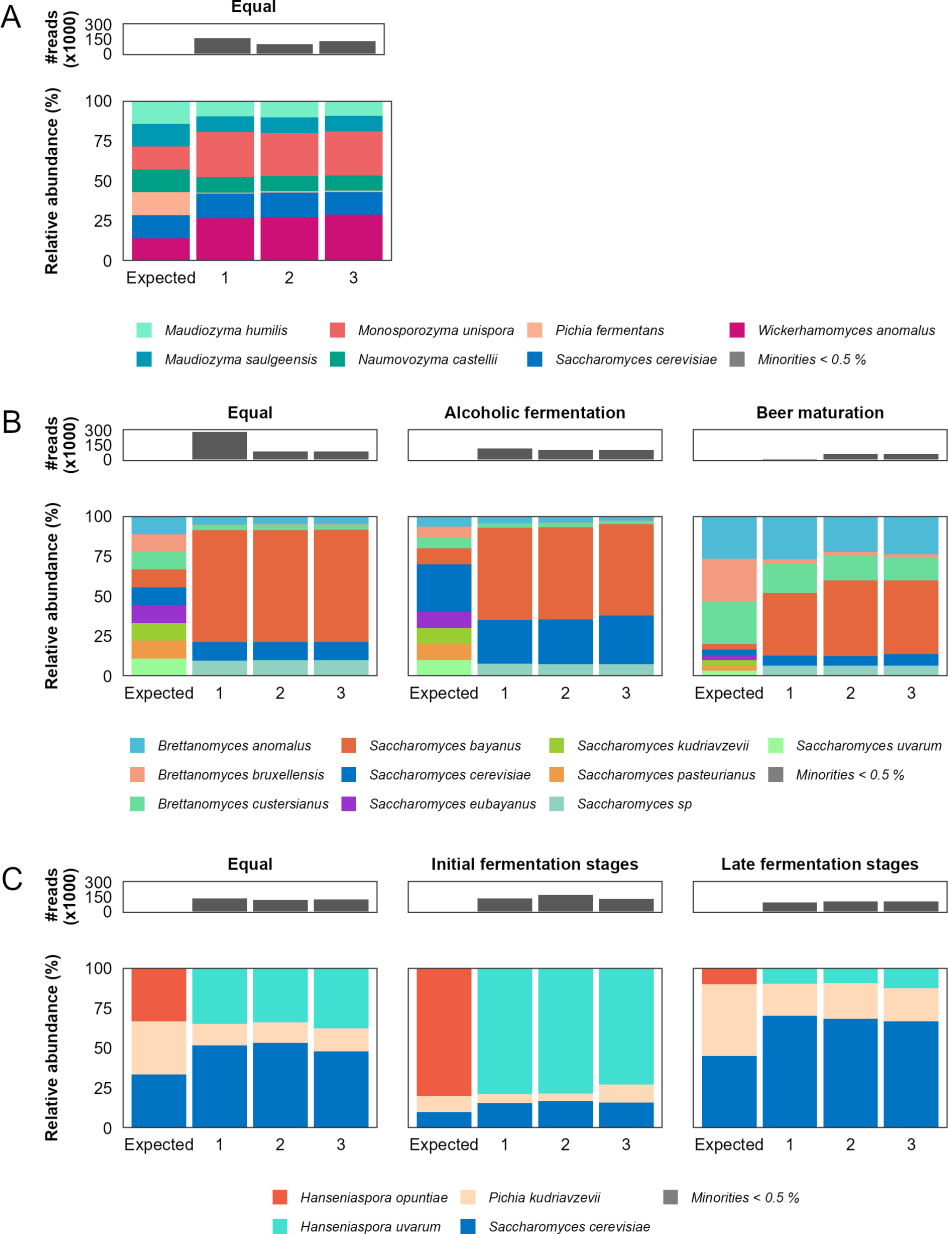
Microbial composition of the sourdough (**A**), lambic beer (**B**), and cocoa (**C**) DNA-based mock communities, depicting both the expected and obtained relative abundances. Sample numbers 1, 2, and 3 represent the different replicates.

A total of seven different DbMCs, representing common yeast species present in the microbial communities of various food fermentation processes, namely those of sourdough, lambic beer, and cocoa, were constructed (Table 1) and analyzed in triplicate. The results obtained for the three replicates of each DbMC were similar, showing good reproducibility of the PCR amplification and sequencing methodology employed (Fig. 1).

**TABLE 1:** Yeast strains and composition of the DNA-based mock communities used in the study^*a*^

Strain	Isolation source	DNA (%)		
		Sourdough equal		
*Maudiozyma humilis* VUB-H061-Y3	Household sourdough (IMDO-VUB)	14		
*Maudiozyma saulgeensis* VUB-H029-Y1	Household sourdough (IMDO-VUB)	14		
*Monosporozyma unispora* MUCL 51234	Bakery sourdough (MUCL)	14		
*Naumovozyma castellii* VUB-H022-Y10	Household sourdough (IMDO-VUB)	14		
*Saccharomyces cerevisiae* MUCL 51207	Bakery sourdough (MUCL)	14		
*Pichia fermentans* WM_6w_t18w_c_Y7	Laboratory sourdough (25)	14		
*Wickerhamomyces anomalus* MUCL 51207	Bakery sourdough (MUCL)	14		

		Lambic beer equal	Lambic beer alcoholic fermentation	Lambic beer maturation
*Brettanomyces anomalus* B43	Lambic beer (IMDO-VUB)	11	7	27
*Brettanomyces bruxellensis* B28	Lambic beer (IMDO-VUB)	11	7	27
*Brettanomyces custersianus* B36	Lambic beer (IMDO-VUB)	11	7	27
*Saccharomyces cerevisiae* MUCL 30006	Beer (MUCL)	11	30	3
*Saccharomyces bayanus* MUCL 27813	Beer (MUCL)	11	10	3
*Saccharomyces eubayanus* CBS 12357	Tree (CBS)	11	10	3
*Saccharomyces kudriavzevii* MUCL 46198	Plant (MUCL)	11	10	3
*Saccharomyces pasteurianus* MUCL 31496	NA (MUCL)	11	10	3
*Saccharomyces uvarum* MUCL 27835	NA (MUCL)	11	10	3

		Cocoa equal	Cocoa early fermentation stages	Cocoa late fermentation stages
*Hanseniaspora opuntiae* Y195	Cocoa fermentation	33	80	10
*Saccharomyces cerevisiae* Y33	Cocoa fermentation	33	10	45
*Pichia kudriavzevii* Y42	Cocoa fermentation	33	10	45

^*a*^ MUCL, Agro-food and Environmental Fungal Collection at the Belgian Coordinated Collections of Microorganisms; IMDO-VUB, Research Group of Industrial Microbiology and Food Biotechnology; and NA, not available.

##### Sourdough DbMC

All species included in the DbMC were retrieved, corresponding to a precision, recall, and F1 score of 1 (Fig. 1A and Table 2). The divergence between the expected composition and the obtained one was 0.27, as some species were found in relative abundances different from the expected ones. Specifically, the obtained relative abundance of *Saccharomyces cerevisiae* was very similar to the expected one (on average, 0.6 ± 0.7 percentage points [%pt] more). In the case of *Maudiozyma humilis*, *Maudiozyma saulgeensis*, and *Naumovozyma castellii*, their relative abundances were slightly below the expected ones (on average, 4.6 ± 0.2 %pt less). In contrast, *Monosporozyma unispora* and *Wickerhamomyces anomalus* were found at a higher relative abundance than the expected ones (on average 13.3 ± 0.8 %pt more). Finally, the relative abundance of *Pichia fermentans* was on average 0.8% ± 0.1%, almost 20 times lower than the expected one.

**TABLE 2:** Metrics describing the deviations obtained from the expected composition of the DNA-based mock communities^*a*^

DNA-based mock community	Divergence	False negatives	False positives	True positives	Precision	Recall	F1 score
Sourdough equal	0.2743 ± 0.0051	0	0	7	1	1	1
Lambic beer equal	0.6915 ± 0.0006	4	1	5	0.83	0.56	0.67
Lambic beer alcoholic fermentation	0.5527 ± 0.0016	4	1	5	0.83	0.56	0.67
Lambic beer maturation	0.5073 ± 0.3595	4	1	5	0.83	0.56	0.67
Cocoa equal	0.5321 ± 0.0063	1	1	2	0.67	0.67	0.67
Cocoa early fermentation stages	0.8318 ± 0.0228	1	1	2	0.67	0.67	0.67
Cocoa late fermentation stages	0.3391 ± 0.0081	1	1	2	0.67	0.67	0.67

^*a*^ The divergence was calculated as the Bray-Curtis distances between the expected and the observed relative abundance. FN, false negatives, the number of expected taxa that were not recovered; FP, false positives, the number of recovered taxa that were not expected; TP, true positives, the number of recovered taxa that were expected; precision, TP/(TP + FP); recall, TP/(TP + FN); and F1 score, 2TP/(2TP + 2FP + FN).

##### Lambic beer DbMCs

The composition of the lambic beer DbMCs was determined with a precision of 0.83, a recall of 0.56, and an F1 score of 0.67 in all cases, and a divergence of, on average, 0.69, 0.55, and 0.51 in the case of the lambic beer equal, lambic beer alcoholic fermentation, and lambic beer maturation DbMCs, respectively (Table 2). A precision, recall, and F1 score lower than 1 could be explained by the fact that the species *Saccharomyces eubayanus*, *Saccharomyces kudriavzevii*, *Saccharomyces pasteurianus*, and *Saccharomyces uvarum* were not found in any of the lambic beer DbMCs (Fig. 1B). Remarkably, 10.0% ± 0.0%, 7.7% ± 0.1%, and 6.3% ± 0.2% of the reads of the lambic beer equal, lambic beer alcoholic fermentation, and lambic beer maturation DbMCs, respectively, were assigned to *Saccharomyces* sp. Blastn searches using the ASVs assigned to this taxonomic unit as a query resulted in the alignment of seven out of eight ASVs to *S. kudriavzevii,* with at least 98.4% sequence identity and 100% coverage. In contrast, the eighth ASV aligned to *S. kudriavzevii* and *Saccharomyces paradoxus* with a sequence identity of 95.8% and a coverage of 100%. As was the case for the sourdough DbMC, *S. cerevisiae* was found at a similar relative abundance to the expected ones in the lambic beer equal (on average 11.4% ± 0.1% vs 11.1%) and the lambic beer alcoholic fermentation DbMCs (on average 28.7% ± 1.4% vs 30.0%). However, this species was found at a relative abundance of 6.5% ± 0.4%, compared to the expected 3.3% in the lambic beer maturation DbMCs. *Saccharomyces bayanus* was found at a higher relative abundance than those expected in the lambic beer equal (70.1% ± 0.1% vs 11.1%), lambic beer alcoholic fermentation (57.4% ± 0.2% vs 10.0%), and lambic beer maturation (44.2% ± 3.6% vs 3.3%) DbMCs. Furthermore, the *Brettanomyces* species were all underrepresented in the three lambic beer DbMCs, with the case of *Brettanomyces bruxellensis* being the most remarkable one (relative abundances lower than 2.4% in all cases).

##### Cocoa DbMCs

The cocoa DbMCs were described with a precision of 0.67, a recall of 0.67, and an F1 score of 0.67 in all cases, with divergences of 0.53, 0.83, and 0.34 in the case of the cocoa equal, cocoa early fermentation stages, and cocoa late fermentation stages DbMCs, respectively (Table 2). These values were the result of incorrect identification of the *Hanseniaspora* species, leading to higher divergences for the DbMCs with higher expected relative abundances for *Hanseniaspora*. Specifically, reads from *Hanseniaspora opuntiae* were identified as *Hanseniaspora uvarum*. Nevertheless, its relative abundances were very similar to the expected ones in the cocoa equal (35.5% ± 1.7% vs 33.3%), cocoa early fermentation stage (75.5% ± 2.8 vs 80%), and cocoa late fermentation stage (10.6% ± 1.4% vs 10%) DbMCs. *S. cerevisiae* was found with a relative abundance 1.5 times higher than the expected one in all cocoa DbMCs (Fig. 1C). In contrast, *Pichia kudriavzevii* was found with a relative abundance that was 2.5, 1.4, and 2.1 times lower than the expected ones for the equal cocoa, cocoa early fermentation stage, and cocoa late fermentation stage DbMCs, respectively.

### Fermented food samples

**Fig 2:**
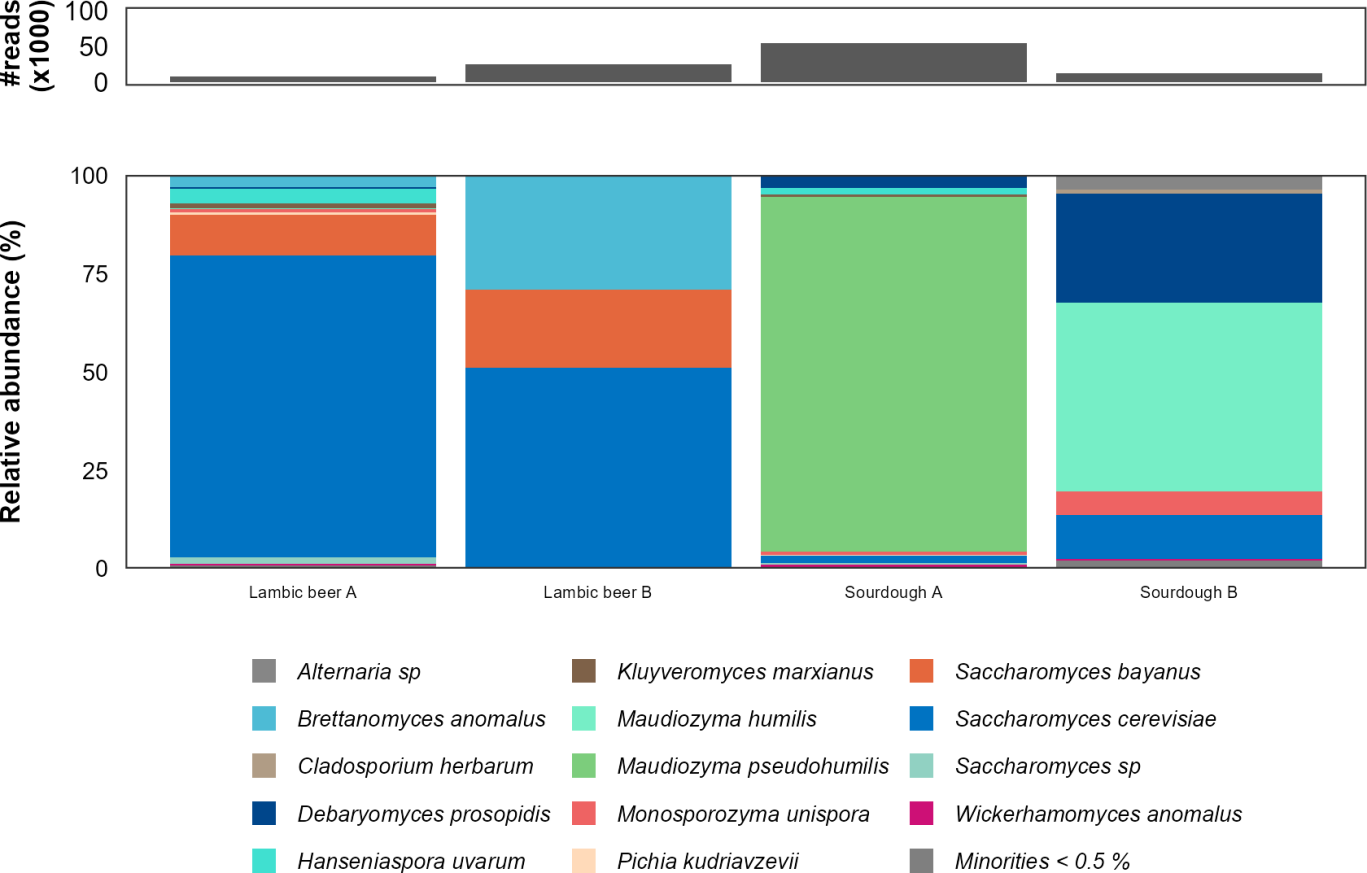
Fungal composition of the fermented food samples. Lambic beer A was taken after 5 days of barrel fermentation, and Lambic beer B was taken after 3 months. Sourdough A was a 3-year-old bakery rye sourdough, and Sourdough B was a 9-year-old bakery wheat sourdough. “Minorities < 0.5%” included species with a relative abundance < 0.5%.

When applying the developed method to the fermented food samples, a high diversity of yeast species was retrieved (Fig. S1B). However, most of the species were present with a relative abundance lower than 0.5% (combined in the “Minorities < 0.5%” category; Fig. 2). The lambic beer samples, Lambic beer A and B, were both mostly inhabited by *S. cerevisiae* (relative abundance of 76.8% and 50.8%, respectively), *S. bayanus* (10.3% and 19.8%, respectively), and *Brettanomyces anomalus* (3.0% and 29.1%, respectively). The sourdough samples displayed different yeast compositions, with Sourdough A containing *Maudiozyma pseudohumilis* (90.1%), *Debaryomyces prosopidis* (3.0%), and *S. cerevisiae* (1.9%) and Sourdough B containing *Ma. humilis* (47.9%), *D. prosopidis* (27.7%), and *S. cerevisiae* (11.2%). In the sourdough samples, two filamentous fungi were retrieved at low relative abundances, namely, *Alternaria* sp. and *Cladosporium herbarum*.

## DISCUSSION

In the present study, the potential of an amplicon-based metabarcoding method targeting the full ITS region to reveal the yeast diversity of fermented food samples was investigated. Up to now, culture-independent approaches revealing the yeast diversity in food fermentations have been performed using short-read sequencing, targeting a small region of the fungal rRNA operon, resulting in an identification that was limited to the genus level (22, 25, 27, 36–38). However, the contribution to food fermentation processes of different species within a genus might differ. Therefore, targeting a larger region that would allow species-level identification was needed in the field.

Thanks to PacBio’s long-read HiFi sequencing technology, in combination with PacBio’s Kinnex library construction strategy, longer amplicons can be sequenced in a cost-efficient manner, providing a higher level of taxonomic resolution compared to previous methods. While the PacBio-based method has already been used to sequence the full ITS region to investigate fungal diversity, those studies focused, in most cases, on filamentous fungi and not on yeasts (21, 31, 33–35). Whether this method would be applicable in the field of food fermentation, in which yeasts are key microorganisms (1–3), remained unclear.

As a first step, seven DNA-based mock communities were composed, representing different stages of sourdough, lambic beer, and cocoa fermentation processes. These DbMCs were composed of different species of the same genus, including *B. anomalus, B. bruxellensis, Brettanomyces custersianus*, *Ma. humilis*, *Ma. saulgeensis*, *Mo. unispora*, *N. castelli, S. cerevisiae*, *P. fermentans, P. kudriavzevii*, and *W. anomalus*. Overall, species-level identification was successfully obtained, with two exceptions, which align with current knowledge in the field. *Hanseniaspora opuntiae* was misidentified as *H. uvarum* due to their known close genetic relationship (39). Also, a limited resolution was observed within the *Saccharomyces* genus, also due to the close genetic relationship among species in this genus (40). Additionally, *Saccharomyces* species are also known to produce natural hybrids, which are frequently found in fermented foods such as beers (41). Therefore, when analyzing samples from *Saccharomyces*-rich niches, the results should be interpreted with caution, as the *Saccharomyces* complexity might not be fully captured. Moreover, further research could tackle the search for other genomic regions that allow for species-level identification of *Saccharomyces* and *Hanseniaspora* species and ideally for species of other genera relevant to fermented foods.

The lack of species-level resolution or species misidentifications, as for the specific taxa discussed above, could be solved by targeting longer regions, such as the full rRNA region, spanning the 18S rRNA gene, ITS1, the 5.8S rRNA gene, ITS2, and the 28S rRNA gene (42, 43). However, such an approach would imply other challenges. Although the length of the expected amplicon, 6 kb, fits perfectly within PacBio’s HiFi and Kinnex sequencing strategies, well-curated, publicly available databases containing the full operon are currently not available. This could be overcome by the construction of custom databases (42, 43). However, the need to construct such custom databases as well as perform regular updates could prevent this strategy from becoming a routine strategy in microbiology laboratories unspecialized in advanced bioinformatics. The launch of the Ribosomal Operon Database (44) might change this scenario, although a regular update of such a recent database cannot be taken for granted, which, given the regular reclassifications of yeasts in recent years, is crucial for accurate yeast identification (45–49).

In addition to accurate species-level identification, the quantitative estimation of the fungal composition, expressed as relative abundance, should be as accurate as possible to obtain a good representation of the real community. However, this is not always possible, as different biases play a role that are difficult (if not impossible) to avoid. In particular, the lower-than-expected relative abundances of *Pichia* species in the sourdough and cocoa DbMCs are in line with previous studies (22, 25). Due to the short ITS regions for both *Pichia* and *Brettanomyces* (approximately 400–500 bp compared to 700–800 bp for *Saccharomyces*), the underestimation of *Brettanomyces* species in the lambic beer DbMCs might have been caused by the same effects. However, further investigation is needed, as length biases do not seem to have a great impact on the relative abundances when targeting either the ITS1 or ITS2 region (12). In addition, the different copy numbers of the ITS region in different yeasts might have introduced a bias during the PCR amplification. As information on yeast rRNA region copy numbers remains scarce and seems to be strain-dependent (44), fungal community profiling should be considered as semi-quantitative, rather than quantitative. This highlights the need for long-read whole-genome sequences, curated and deposited in a public database, to enable accurate copy number determination at the species or strain level, moving toward a more quantitative profiling of fungal communities, irrespective of their habitat.

Given the promising results of the DbMCs, the use of the newly developed method on sourdough and lambic beer samples resulted in a confident description of the fungal diversity at the species level using a culture-independent approach. The species corresponded with those generally described for these matrices using a culture-dependent approach (50, 51). Special attention should be paid to *S. bayanus* for lambic beer samples, as other *Saccharomyces* species might also be present. Furthermore, the high level of UFGR in the sourdough samples could be explained by the presence of the ITS region in plants, which, in some cases, might be similar enough to allow primer hybridization, as described before (12, 52). Therefore, when using this method in cereal-rich matrices, a high sequencing depth should be pursued to have a sufficient number of sequence reads to be able to reliably describe the full yeast diversity after the removal of the UFGR, as was the case in the present study. Moreover, optimization of the centrifugation steps before DNA extractions to ensure that most of the plant cells are removed could be considered.

Finally, biases introduced during DNA extraction were not assessed in the current study, as the same DNA extraction methods were applied to make the DNA-based mock communities that were previously successfully applied for different fermented food matrices (24, 25, 36, 37, 53, 54). Further optimization of DNA extraction methods has the potential to improve the community composition analysis.

In conclusion, amplicon-based metabarcoding targeting the full ITS region using the PacBio HiFi sequencing technology is a promising method to unravel yeast diversity in fermented food samples, achieving a better taxonomic resolution compared to the commonly used Illumina-based methodologies. However, further research is needed to find ways to improve the identification at the species level in environments rich in *Saccharomyces* and *Hanseniaspora* species.

## MATERIALS AND METHODS

### DNA-based mock communities

To critically assess the methodology developed during the present study that aimed to investigate yeast diversity through amplicon-based metabarcoding of the ITS1-2 region, different DbMCs were constructed representing strains of species that play an important role in food fermentation processes.

#### Strains, growth conditions, and yeast genomic DNA extraction

All yeast strains used in the present study (Table 1) were stored at −80°C in cryovials containing yeast extract-peptone-glucose (YPG; Oxoid, Basingstoke, Hampshire, UK) cultures (55), supplemented with 25% (vol/vol) glycerol (Sigma-Aldrich, Saint-Louis, MO, USA) as cryoprotectant, as part of the laboratory collection of the Research Group of Industrial Microbiology and Food Biotechnology (IMDO-VUB). Each yeast strain was streaked on YPG agar medium supplemented with 200 ppm of chloramphenicol (Sigma-Aldrich) and incubated at 30°C for 48 h. A single colony of each strain was transferred to 10 mL of liquid YPG medium and incubated at the same temperature for 24 h. Yeast cell pellets were obtained by centrifugation of 2 mL of the latter cultures at 21,100 × *g* for 5 min. Yeast genomic DNA was extracted from these cell pellets using 600 µL of enzymatic lysis buffer with lyticase (200 U; Sigma-Aldrich), zymolyase (15 U; G-Biosciences, Saint Louis, MO, USA), and proteinase K (60 mAnsonU; Merck, Darmstadt, Germany) as described before (24). DNA purification was performed using a DNeasy Blood and Tissue kit (Qiagen, Hilden, Germany). The DNA concentrations were measured by fluorimetry (Qubit; Thermo Fisher Scientific, Waltham, MA, USA).

#### Composition of the DbMCs

A general DbMC, already available in the IMDO-VUB research group, composed of 17 strains of fermented food-related yeast species (Table S1), was used to evaluate the success of the PCR amplification (Temperature gradient section).

A total of seven different DbMCs, including the most abundant yeast species present in the microbial communities of various food fermentation processes, namely those of sourdough, lambic beer, and cocoa, were constructed (Table 1). In the case of sourdough, one DbMC containing equal concentrations of DNA of seven common sourdough yeast species (51, 56) was constructed. In the case of lambic beer, three DbMCs were considered, namely one DbMC with equal concentrations of DNA of three *Brettanomyces* and six *Saccharomyces* species, one DbMC representing the alcoholic fermentation phase of lambic beer that is rich in *Saccharomyces* species, and one DbMC representing the maturation phase of lambic beer that is rich in *Brettanomyces* species (50). In the case of cocoa, three DbMCs were considered, namely, one DbMC with equal concentrations of DNA of *H. opuntiae*, *S. cerevisiae,* and *P. kudriavzevii*, one DbMC representing the early stage of the cocoa fermentation process that is rich in *Hanseniaspora* species (4), and one DbMC representing the late stage of the cocoa fermentation process that is rich in *S. cerevisiae* and *Pichia* species (4). Each DbMC was constructed by pooling the strains’ DNA (Table 1) to get a final total DNA concentration of 1.5 ng/µL.

### Fermented food samples

#### Sampling

To assess the method’s validity in fermented food samples, two sourdough samples and two lambic beer samples were analyzed. However, no recently collected cocoa samples were available at the time of the study. The sourdough samples were both spontaneous Type 1 sourdoughs originating from two artisan bakeries, with Sourdough A being a 3-year-old rye sourdough and Sourdough B being a 9-year-old wheat sourdough. Cell pellets were collected by diluting the sourdoughs 1:10 with sterile peptone saline (0.1% bacteriological peptone [Oxoid] and 0.85% NaCl [Merck]) as described before (25). After mixing to get a homogenous sample, a two-step centrifugation was performed. First, the homogenous samples were centrifuged at 1,000 × *g* for 5 min. Second, the flour debris-free supernatants were centrifuged at 5,000 × *g* for 20 min to collect the cell pellet. The lambic beer samples consisted of samples taken during the production of lambic beer, with Lambic beer A taken after 5 days of barrel fermentation and Lambic beer B taken after 3 months of barrel fermentation. The cell pellets were collected by a centrifugation step at 5,000 × *g* for 20 min (21). In both cases, the cell pellets were stored at −20°C until further DNA extraction.

#### DNA extraction from fermented food samples

Extraction of total DNA from the cell pellets was performed as described for sourdough (25) and lambic beer samples (22). Briefly, in both cases, a combination of both a bacterial lysis solution (300 µL of bacterial lysis buffer with 100 U mutanolysin [Sigma-Aldrich] and 320 kU of lysozyme [Merck]) and a yeast lysis solution (600 µL of yeast lysis buffer with 15 U zymolyase [G-Biosciences] and 200 U lyticase [Sigma-Aldrich]) was used to lyse the bacterial and yeast cells. Furthermore, mechanical lysis was performed using UV-C-irradiated glass beads (Sigma-Aldrich). Then, protein digestion was performed with 40.0 μL of a 20.0% (m/m) sodium dodecyl sulfate (Sigma-Aldrich) solution and 50.0 μL of proteinase K solution (2.0 mg/mL of proteinase K; Merck) per sample. In the case of the sourdough samples, they were further incubated in a 5.0 M NaCl (Merck) solution and a 10.0% (m/m) cetyltrimethylammonium bromide (Merck) solution. Finally, DNA was purified using chloroform-phenol-isoamyl alcohol solution and a DNeasy Blood and Tissue kit (Qiagen). The DNA concentrations were measured by Qubit fluorimetry (Thermo Fisher Scientific).

### PCR

#### Selection of the primer sets

From all primers described to target the yeast rRNA operon (27, 57, 58) and, in particular, from those that allow for amplifying the full internal transcribed region, two pairs of primers were selected. Specifically, primers BITS (5′-ACCTGCGGARGGATCA-3′) and ITS1 (5′-TCCGTAGGTGAACCTGCGG-3′) were selected as forward primers, and primer ITS4 (5′-TCCTCCGCTTATTGATATGC-3′) was selected as reverse primer. The BITS primer, together with B58S3 as a reverse primer, has previously been used to target the fungal ITS1 region to describe the fungal diversity of food fermentation processes using Illumina amplicon-based metabarcoding (22, 25, 27, 36–38). The primers ITS1 and ITS4 are generally used to amplify the ITS1-2 region to identify yeast isolates in a culture-dependent approach (22, 25, 37, 38, 59, 60). The primers were tagged with Kinnex adaptors and 5′ sample-specific barcodes (Integrated DNA Technologies, Leuven, Belgium; Table S2) to allow for multiplexed sequencing, according to the manufacturer’s instructions (Pacific Biosciences, Menlo Park, CA, USA).

#### Temperature gradient

PCR assays were performed using KAPA HiFi DNA Polymerase (Hot Start and Ready Mix; Roche, Basel, Switzerland) based on the PCR assay described for 16S rRNA gene amplification for PacBio HiFi sequencing (24), but with a different annealing temperature. These PCR amplifications were done using the general DbMC as a DNA template. Specifically, per sample, 1.5 µL of DNase and RNase-free water (VWR International, Radnor, PA, USA), 12.5 µL of 2× KAPA HiFi HotStart ReadyMix (Roche), 3 µL of the forward primer, 3 µL of the reverse primers (2.5 µM each), and 5 µL of template DNA (1.5 ng/µL) were mixed. To assess the most optimal annealing temperature, PCR assays were performed applying a temperature gradient using a TProfessional Basic Gradient thermocycler (BioMetra, Göttingen, Germany) with values 52.0°C, 53.0°C, 54.2°C, 55.4°C, 56.0°C, and 57.0°C as annealing temperatures, in combination with an initial denaturation at 95°C for 3 min, followed by 27 cycles of denaturation at 95°C for 30 s, annealing for 30 s, and extension at 72°C for 60 s. The final PCR assays consisted of 3 min of denaturation at 95°C, followed by 27 cycles of denaturation at 95°C for 30 s, annealing at 52°C for 30 s, and extension at 72°C for 60 s. The PCR amplicons obtained were visualized through gel electrophoresis using 1.5% (m/v) agarose gels and performed at 100 V for 1 h. PCR amplicon purification was performed using a Wizard Plus SV Mini-preps DNA purification system (Promega, Madison, WI, USA). PCR assays for all DbMCs and food samples were performed in triplicate with sample-specific barcodes (Table S3).

### PacBio HiFi long-read sequencing

After PCR amplification with the sample-specific barcoded primers, sequencing library preparation was done following PacBio’s workflow (“Procedure & checklist—preparing Kinnex libraries from 16S rRNA amplicons,” PacBio Document 103-238-800). Briefly, amplicons were pooled, and a Kinnex PCR was performed, during which Kinnex terminal adaptors were added to the amplicons to enable concatenation. Next, amplicons were concatenated using the Kinnex enzyme and ligase. Finally, SMRTbell adapters were ligated to generate circular DNA templates suitable for circular consensus sequencing. The obtained SMRTbell library was sequenced on a Revio platform (Pacific Biosciences) at the VIB Nucleomics Core Facility (Leuven, Belgium).

### Data processing

The reads obtained from the Revio platform were deconcatenated using Skera (v1.3.0, PacBio), and individual amplicons were demultiplexed using Lima (v2.12.0, Pacific Biosciences), with a minimal length cutoff set at 50 bp to avoid removing any short ITS sequences. Demultiplexed reads were further processed using RStudio (version 4.2.1 [61]), and amplicon sequence variants were obtained using the DADA2 package (version 1.26.0 [62]). The filtering parameters used were minQ = 2, minLen = 300, maxLen = 1,000, maxN = 0, and maxEE = 5. Taxonomy was assigned using the fungal-specific version of the UNITE database (v10.0 [63]) using a minBoot = 80. Reads not identified at the genus level (“unidentifiable fungal genus reads”) were filtered out. ASVs were grouped per species, and relative abundances of each species per sample (number of reads assigned to one species in one sample/total number of reads of that sample × 100) were calculated, including those species with a relative abundance below 0.5% under the category “Minorities < 0.5%.” Furthermore, the UFGRs were used as queries for blastn searches using the core_nt database of the National Center for Biotechnology Information (July 2025).

Rarefaction curve analysis was performed using the R package vegan (v2.6-4 [64]). The divergence rate was calculated as the Bray-Curtis distance between the expected and the observed relative abundance using the same R package. The expected relative abundance was calculated based on the percentage of DNA of each species included in each DbMC, whereas the observed relative abundance was the one obtained after processing the sequence data. The number of false negatives (FNs) was defined as the number of expected taxa that were not recovered, and the false positives (FPs) were defined as the number of recovered taxa that were not expected. Furthermore, the number of true positives (TPs) was defined as the number of recovered taxa that were expected. Finally, the precision of the method was calculated as TP/(TP + FP), the recall as TP/(TP + FN), and the F1 score as 2TP/(2TP + 2FP + FN) (12).

## Data Availability

The sequence reads are available at the European Nucleotide Archive of the European Bioinformatics Institute (ENA/EBI) under the BioProject accession number PRJEB101075.

## SUPPLEMENTAL MATERIAL

Supplemental figure and tables spectrum.00060-26-s0001.pdf Tables S1 to S4 and Figure S1.

ASM does not own the copyrights to Supplemental Material that may be linked to, or accessed through, an article. The authors have granted ASM a non-exclusive, world-wide license to publish the Supplemental Material files. Please contact the corresponding author directly for reuse.
