# Supplementary material for: Unraveling yeast diversity in food fermentation using ITS1-2 amplicon-based metabarcoding
Source: Microbiol Spectr. 2026 Jul 6;14(8):e00060-26. doi: 10.1128/spectrum.00060-26 (PMC13435807; doi:10.1128/spectrum.00060-26)

Supplementary material

Supplementary Table S1. Composition of the general DNA-based mock community.

Species	DNA (%)
Brettanomyces anomalus	5.9
Brettanomyces bruxellensis	5.9
Brettanomyces custersianus	5.9
Debaryomyces hansenii	5.9
Hanseniaspora opuntiae	5.9
Hanseniaspora uvarum	5.9
Kluyveromyces marxianus	5.9
Maudiozyma bulderi	5.9
Maudiozyma humilis	5.9
Monosporozyma unispora	5.9
Nakaseomyces glabratus	5.9
Pichia kudriavzevii	5.9
Saccharomyces cerevisiae	5.9
Saccharomyces kudriavzevii	5.9
Torulaspora delbreuckii	5.9
Wickerhamomyces anomalus	5.9
Yarrowia lipolytica	5.9

Supplementary Table S2. Primer sequences (ITS1 and ITS4) tagged with Kinnex adaptors (underlined) and 5' sample-specific barcodes (bold).

Primer	Sequence (5'-3')
04-BITS	CTACACGACGCTCTTCCGATCT**GTACTGTCAG**ACCTGCGGARGGATCA
05-BITS	CTACACGACGCTCTTCCGATCT**ACGTGCAGAT**ACCTGCGGARGGATCA
06-BITS	CTACACGACGCTCTTCCGATCT**GACTATGACG**ACCTGCGGARGGATCA
01-ITS1	CTACACGACGCTCTTCCGATCT**GATCGAGTCA**TCCGTAGGTGAACCTGCGG
02-ITS1	CTACACGACGCTCTTCCGATCT**AGATCGCATG**TCCGTAGGTGAACCTGCGG
03-ITS1	CTACACGACGCTCTTCCGATCT**AGACTAGCGT**TCCGTAGGTGAACCTGCGG
04-ITS1	CTACACGACGCTCTTCCGATCT**GTACTGTCAG**TCCGTAGGTGAACCTGCGG
06-ITS1	CTACACGACGCTCTTCCGATCT**GACTATGACG**TCCGTAGGTGAACCTGCGG
07-ITS1	CTACACGACGCTCTTCCGATCT**GACGCATAGT**TCCGTAGGTGAACCTGCGG
08-ITS1	CTACACGACGCTCTTCCGATCT**AGCATGTACG**TCCGTAGGTGAACCTGCGG
09-ITS1	CTACACGACGCTCTTCCGATCT**GTCGCACGAT**TCCGTAGGTGAACCTGCGG
10-ITS1	CTACACGACGCTCTTCCGATCT**AGTGCGATCG**TCCGTAGGTGAACCTGCGG
19-ITS4	AAGCAGTGGTATCAACGCAGAG**CAGTATGAGC**TCCTCCGCTTATTGATATGC
20-ITS4	AAGCAGTGGTATCAACGCAGAG**ACGATGACGT**TCCTCCGCTTATTGATATGC
39-ITS4	AAGCAGTGGTATCAACGCAGAG**ACGCACGTAC**TCCTCCGCTTATTGATATGC
40-ITS4	AAGCAGTGGTATCAACGCAGAG**CAGTAGCGTC**TCCTCCGCTTATTGATATGC
41-ITS4	AAGCAGTGGTATCAACGCAGAG**ACTGCAGCAC**TCCTCCGCTTATTGATATGC
42-ITS4	AAGCAGTGGTATCAACGCAGAG**TCACGACGAC**TCCTCCGCTTATTGATATGC
43-ITS4	AAGCAGTGGTATCAACGCAGAG**CGAGTCTAGC**TCCTCCGCTTATTGATATGC
44-ITS4	AAGCAGTGGTATCAACGCAGAG**CAGCAGTGAC**TCCTCCGCTTATTGATATGC

Supplementary Table S3. Samples (DNA-based mock communities and actual samples) used in the present study, the barcode-primer combinations, and the number of original sequenced reads and retained reads after each data processing step. ccs, circular consensus sequence.

Sample	Forward primer	Reverse primer	Number of sequence reads			
			Original (ccs)	Primer removal	Quality filtering	Denoising
Sourdough equal 1	01-ITS1	44-ITS4	186343	181301	167840	167551
Sourdough equal 2	12-ITS1	39-ITS4	117557	111814	103564	103368
Sourdough equal 3	08-ITS1	44-ITS4	155641	152234	141318	140903
Lambic beer equal 1	03-ITS1	43-ITS4	348099	341611	310800	310563
Lambic beer equal 2	06-ITS1	44-ITS4	104465	100495	91552	91441
Lambic beer equal 3	10-ITS1	43-ITS4	101010	96037	87404	87342
Lambic beer alcoholic fermentation 1	02-ITS1	43-ITS4	141013	136677	124331	124276
Lambic beer alcoholic fermentation 2	12-ITS1	40-ITS4	128998	122849	111657	111578
Lambic beer alcoholic fermentation 3	09-ITS1	43-ITS4	118894	115626	104939	104868
Lambic beer maturation 1	02-ITS1	44-ITS4	10361	9973	9268	9221
Lambic beer maturation 2	06-ITS1	43-ITS4	69090	65993	61070	60979
Lambic beer maturation 3	09-ITS1	44-ITS4	70720	68414	63357	63269
Cocoa equal 1	01-ITS1	43-ITS4	152187	147622	135334	135235
Cocoa equal 2	04-ITS1	44-ITS4	140041	133929	123095	123051
Cocoa equal 3	08-ITS1	43-ITS4	144276	141314	130006	129925
Cocoa early fermentation stages 1	12-ITS1	41-ITS4	154665	147805	136116	136063
Cocoa early fermentation stages 2	03-ITS1	44-ITS4	189032	185193	170470	170391
Cocoa early fermentation stages 3	07-ITS1	43-ITS4	146225	143053	131783	131746
Cocoa late fermentation stages 1	12-ITS1	42-ITS4	105496	100985	92620	92578
Cocoa late fermentation stages 2	04-ITS1	43-ITS4	118932	113936	104485	104463
Cocoa late fermentation stages 3	07-ITS1	44-ITS4	117751	115005	105640	105580
Sourdough A	07-ITS1	19-ITS4	76731	74689	66519	66435
Sourdough B	07-ITS1	20-ITS4	38114	36965	32790	32662
Lambic beer A	08-ITS1	19-ITS4	9718	9425	8054	8007
Lambic beer B	08-ITS1	20-ITS4	29593	28861	25285	25264

Supplementary Table S4. Relative abundances of the unidentifiable fungal genus reads.

Sample	Unidentifiable fungal genus reads (%)
Sourdough equal 1	0
Sourdough equal 2	0.003
Sourdough equal 3	0.001
Lambic beer equal 1	0.001
Lambic beer equal 2	0
Lambic beer equal 3	0
Lambic beer alcoholic fermentation 1	0.003
Lambic beer alcoholic fermentation 2	0
Lambic beer alcoholic fermentation 3	0
Lambic beer maturation 1	0
Lambic beer maturation 2	0
Lambic beer maturation 3	0
Cocoa equal 1	0.086
Cocoa equal 2	0.088
Cocoa equal 3	0.214
Cocoa early fermentation stages 1	0.091
Cocoa early fermentation stages 2	0.098
Cocoa early fermentation stages 3	0.082
Cocoa late fermentation stages 1	0.014
Cocoa late fermentation stages 2	0.024
Cocoa late fermentation stages 3	0.042
Sourdough A	16.668
Sourdough B	60.818
Lambic beer A	0.138
Lambic beer B	0

Supplementary Figure S1. Rarefaction curves of the DNA-abased mock communities (A) and food fermentation samples (B). In (A), full lines represents replicate 1, dashed lines replicate 2, and dotted lines replicate 3.

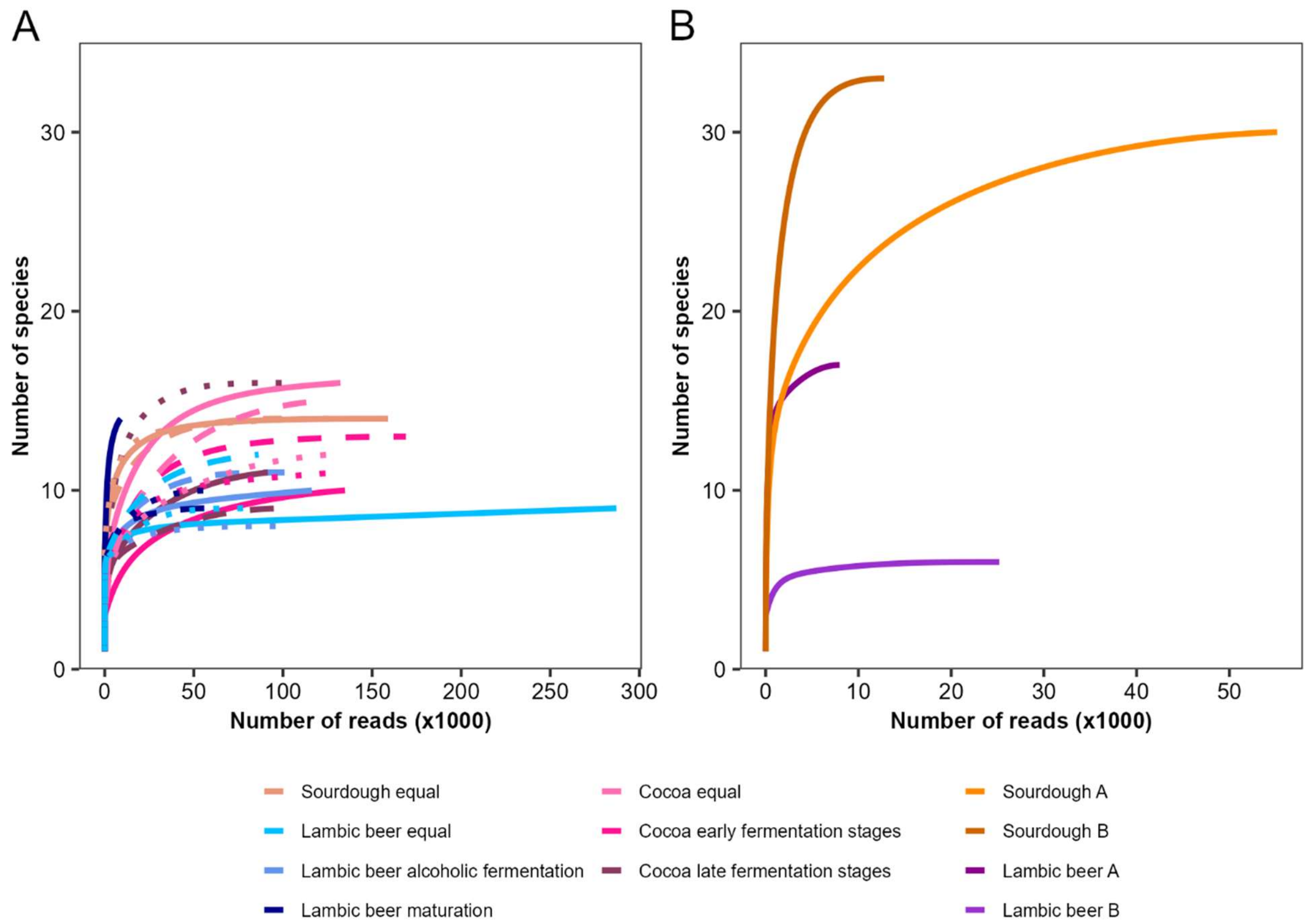

Supplement: Supplementary file 1 [file spectrum.00060-26-s0001.pdf]
